# Effects of 3′,4′-dihydroxyflavonol treatment on Neu N and BDNF and ICAM levels in the striatum of rats subjected to transient bilateral carotid occlusion-induced ischemia–reperfusion

**DOI:** 10.1007/s10863-026-10109-x

**Published:** 2026-05-23

**Authors:** Murat Hoşgör, Ebru Kubra Uzdil, Tugce Aladag, Seda Simsek, Nejat Unlukal, Rasim Mogulkoc, Abdulkerim Kasim Baltaci

**Affiliations:** 1 Faculty of Medicine, Department of Physiology, Selçuk University, 42131 Konya, Turkey; 2 Faculty of Medicine, Department of Histology and Embriology, Selçuk University, 42131 Konya, Turkey

**Keywords:** Cerebral ischemia reperfusion,DiOHF, Neu N, Alpha Tubulin, Beta III Tubulin, Calbindin, BDNF, ICAM

## Abstract

Cerebral infarction is a leading cause of severe long-term disability and functional and cognitive impairment. With the advancement of acute stroke treatment, more patients are now experiencing stroke with varying degrees of impairment. The present study was conducted to determine the effects of 1-week 3,4-dihydroxyflavonol (DiOHF) administration during transient experimental cerebral ischemia–reperfusion (I/R) in rats on NeuN, Tuba1a, Tubb3, and calbindin were evaluated as markers of neuronal phenotype and cytoskeletal organization, while ICAM and BDNF were assessed in relation to inflammatory and neurotrophic processes. Changes in these markers indicate alterations in neuronal marker expression, cytoskeletal integrity, and neurotrophic and inflammatory status. In this study, a total of 28 male Wistar albino rats, aged 10–12 weeks and weighing 300–400 g, were used. 1-Control Group (n = 6): The animals in this group received no anaesthesia or surgical procedures. 2-Sham Group (n = 6): After general anaesthesia was induced in the animals in this group, the carotid artery regions were opened and closed. 3- Ischemia–Reperfusion Group (n = 8): Under general anaesthesia, the carotid arteries of the rats were isolated and ligated for 30 min, followed by ischemia. 4- Ischemia–Reperfusion + DiOHF Group (n = 8): Under general anaesthesia, the carotid arteries of the rats were ligated for 30 min, followed by ischemia. Reperfusion was then allowed. Tuba1A, Tubb3, ICAM and Calbindin were analyzed by real-time PCR, BDNF by a commercial ELISA kit, and NeuN by immunohistochemistry. I/R decreased the levels of Neu N, Tuba1a, Tubb3, calbindin and BDNF in the striatum tissues and increased ICAM levels. DiOHF supplementation halted the decrease in expression level in Tuba1a, Tubb3, Calbindin, BDNF; the Increase in Icam level.Also DiOHF supplementation prevented the decrease in the level of anti-NeuN antibody and led to an increase. The study results revealed that one week of transient I/R in rats suppressed NeuN, TUBA1A, TUBB3, calbindin and BDNF levels, which are important in neuronal phenotype and cytoskeletal organization inflammatory and neurotrophic processes. However, a week DiOHF treatment significantly corrected the distortions caused by I/R.

## Introduction

Stroke is a leading cause of serious long-term disability, as well as functional and cognitive impairment. With advances in acute stroke treatment, stroke now results in varying degrees of disability in a greater number of patients (Virani et al. 2020). Neurogenesis, the process by which new neurons are generated from neural stem cells (NSCs), has been found to continue during prenatal, postnatal, and adult life in both rodents and the human brain (Boldrini et al. 2018). NSCs have been identified in the subgranular zone (SGZ) of the hippocampus and the subventricular zone (SVZ) of the lateral ventricles in the adult mammalian brain (Suh et al. 2009). After cerebral ischemia, NSCs in these two regions tend to proliferate, and the generated neuroblasts alter their destination toward the ischemic region, attempting to repair the brain where they differentiate into mature neurons (Lindvall and Kokaia 2015). Modulating neurogenesis after cerebral ischemia using various therapeutic agents that target the proliferation, migration, and differentiation of newly generated neurons suggests a novel therapeutic strategy for post-stroke recovery (Moskowitz et al. 2010). Focal middle cerebral artery patterns create localized infarction and penumbra, while transient bilateral carotid artery segments lead to widespread forebrain hypoperfusion, delayed distributional degeneration, and selective features in metabolically sensitive distribution centers (Pulsinelli and Brierley 1979). While SGZ and SVZ are classical neurogenic niches, neurogenesis in the present study was assessed indirectly through neuronal and neurogenesis-associated markers in striatal tissues rather than by niche-specific cell quantification.

Flavonoids, an important group of polyphenolic compounds commonly found in the daily diet, have attracted considerable attention due to their antioxidant, anti-inflammatory, and neuroprotective properties. They are classified into six major subclasses: flavonols, flavones, isoflavones, flavanones, flavanols, and anthocyanidins (Manach et al. 2004). Accumulating evidence indicates that flavonoids exert neuroprotective effects by modulating intracellular signaling pathways involved in neuronal survival, differentiation, synaptic plasticity, and the regulation of neurogenesis (Spencer et al. 2009).

3′,4′-Dihydroxyflavonol (DiOHF) is a synthetic flavonol derivative belonging to the flavonoid family that has recently emerged as a promising neuroprotective agent. Experimental studies have demonstrated that DiOHF improves neuronal and cognitive functions under ischemia–reperfusion conditions; however, its effects under transient bilateral carotid occlusion-induced global hypoperfusion conditions have not yet been sufficiently clarified (Aladag et al. 2024). Despite these established antioxidant and neuroprotective properties, the effects of DiOHF on neurogenesis-related markers, neurotrophic factors such as BDNF, and inflammatory molecules including Icam—particularly in the striatum following cerebral ischemia–reperfusion—remain largely unexplored.

The development of newborn neurons derived from neural stem cells (NSCs) proceeds through multiple sequential stages, each characterized by the expression of specific protein markers before functional integration into existing neural circuits (Kuhn et al. 2016). Markers such as Calretinin, NeuN, and Calbindin are widely used to identify newly generated neurons and to assess their maturation and integration within local circuitry (Kozareva et al. 2019). With few exceptions, NeuN is expressed in mature neurons in the adult brain and is therefore considered a reliable indicator of neuronal integrity (Bartkowska et al. 2023). Also NeuN antibodies are a usefl marker used to obtain immunohistochemical images of neuronal structure (Mullen et al. 1992). In adult neurogenesis, neuron-specific class III β-tubulin (Tuj-1) is commonly used as a marker of newly generated, immature postmitotic neurons (Virani et al. 2020).

Cerebral ischemia–reperfusion (I/R), which occurs following the restoration of blood flow after transient ischemia, induces time-dependent impairments in brain structure and function and has been shown to disrupt the expression ofneurogenesis-related markers. Previous studies using flavonoid-based interventions have demonstrated that I/R leads to marked reductions in Calbindin, α-Tubulin, β-Tubulin, and NeuN expression in hippocampal and cortical tissues, whereas flavonoid supplementation such as naringin can partially restore these markers and support neuronal recovery (Keskin et al. 2025). These findings suggest that flavonoids may modulate neuronal and molecular responses following I/R injury, providing a rationale for investigating whether the synthetic flavonol DiOHF exerts similar effects on neuronal marker expression in the present study.

The striatum is the largest component of the basal ganglia. It is responsible for movement control and addictive behaviours. The striatum is a forebrain structure located beneath the cortex and plays a role in regulating motor behaviours as well as responses to rewarding and aversive stimuli (Ernst and Frisén 2015). In adult humans, the presence of neuroblasts in the subventricular zone—but the detection of only a few neuroblasts migrating along the rostral migratory stream or leading to neurons in the olfactory bulb—has raised questions about the fate of these neuroblasts (Bergmann et al. 2015). Previous studies have confirmed neuronal damage in the hippocampus in an ischemia/reperfusion model created following bilateral carotid artery insufficiency (Atlasbaf et al. 2025). In transient global ischemia models, although the hippocampus is considered a vulnerable area, striatal neuronal integrity is also affected through forebrain hypoperfusion, impaired corticostriatal input, decreased neurotrophin transport, and altered metabolic support. Therefore, evaluation of striatal neuronal markers can provide important information about secondary forebrain molecular damage after bilateral carotid hypoperfusion (Baydyuk and Xu 2014). Recent studies have shown that the development, survival, and proper functioning of striatal neurons depend on neurotrophins (Park and Poo 2013). Brain-derived neurotrophic factor (BDNF) plays a crucial role in neuronal survival and synaptic plasticity, which are fundamental processes underlying learning and memory (Binder and Scharfman 2004). BDNF is synthesised in other brain regions such as the cortex, amygdala, and thalamus, and is transported anterogradely to the striatum along axonal processes (Atlasbaf et al. 2025). Since the striatum does not produce BDNF and depends on it, abnormalities in anterograde transport and reduced gene expression from brain regions that supply BDNF to the striatum can lead to neuronal dysfunction and striatal atrophy (Blankenberg et al. 2003).

Intercellular adhesion molecules (ICAMs) consist of five members: ICAM-1 (widely expressed), ICAM-2 (found in leukocytes, platelets, and endothelium), ICAM-3 (found in endothelial cells and leukocytes), ICAM-4 (erythrocytes), and ICAM-5 (brain) (Blankenberg et al. 2003). In a conducted study, immunohistochemistry of ICAM showed that the number of ICAM-1 positive microvessels in the cortex significantly increased on the third day of reperfusion in diabetic rats, whereas no increase was observed in non-diabetic rats (Ding et al. 2005). BDNF attenuates IL-1 beta-induced ICAM-1 mRNA and protein expression (Takeda et al. 2016). The study investigated the neuroprotective effect of fisetin in cerebral ischemia–reperfusion-induced brain injury and the potential role of nuclear factor kappa B signalling. In vitro, fisetin significantly reduced inflammatory mediators, including intercellular adhesion molecule-1, following ischemia/reperfusion induced in cells (Zhang and Cui 2021). The aim of the present study was to determine the effects of 1 week of DiOHF administration on calbindin, alpha–beta-tubulin, ICAM, BDNF and Neu-N levels in striatum tissue in rats after bilateral carotid occlusion induced transient ischemia reperfusion.

## Materials and methods

The study was conducted at the Selçuk University Experimental Medicine Research and Application Centre with the approval of the Ethics Committee (decision date: 29.02.2024, decision number: 2024–14). Authors declarate that all methods were carried out in accordance with relevant guidelines and regulations. A statement to confirm that all methods are reported in accordance with ARRIVE guidelines. Twenty-eight male Wistar albino rats, aged 10–12 weeks and weighing between 300–400 g, were used. The surgical and testing procedures were performed at the Selcuk University Experimental Medicine Research and Application Centre; biochemical analyses were conducted at the Molecular Physiology Laboratory of Selcuk University Faculty of Medicine, and histological analyses were performed at the Histology Laboratory of the same faculty.

Transient global ischemia was induced by ligating the carotid arteries for 30 min, followed by reperfusion. The procedure is specifically defined as transient bilateral carotid artery occlusion (tBCAO). At the end of the experimental period, brain tissues were collected one week after the ischemia–reperfusion procedure, immediately following completion of the 7-day DiOHF treatment protocol. Animals were sacrificed by cervical dislocation. Subsequently, animals were sacrificed by cervical dislocation. Brain tissues were rapidly removed. Striatal tissues (left hemisphere) designated for molecular analyses (Tuba1a, Tubb3, Calbindin, BDNF, and ICAM) were snap-frozen and stored at − 80 °C. For NeuN immunohistochemical analysis, striatal tissues (right hemisphere) were fixed by immersion in freshly prepared 4% paraformaldehyde at + 4 °C (fixative:tissue volume ratio 10:1).

### Groups

In this study, a total of 28 rats were used and divided into the following groups:

1. **Control Group (n = 6):** No anaesthesia or surgical procedures were applied to the animals in this group.
2. **Sham Group (n = 6):** After induction of general anaesthesia, the carotid artery regions were exposed and then closed without ligation. Following the procedure, the animals received an intraperitoneal injection of solvent (1 ml—hazelnut oil and 5% DMSO) for one week.
3. **Ischemia–Reperfusion Group (n = 8):** Under general anaesthesia, the carotid arteries of the rats were isolated and ligated for 30 min to induce ischemia, followed by reperfusion after 30 min.
4. **Ischemia–Reperfusion + DiOHF Group (n = 8):** Under general anaesthesia, ischemia was induced by ligating the carotid arteries for 30 min, after which reperfusion was allowed. Along with reperfusion, the animals received intraperitoneal DiOHF treatment at a dose of 10 mg/kg/day in 1 ml for one week (Zhang and Cui 2021). DiOHF (CAS Number: 6068–78-6, Catalog No. T-601) purchased from INDOFINE Chemical Company, Inc. New Jersey, USA.

### Surgical procedure

General anaesthesia was induced in rats by intraperitoneal administration of a mixture of ketamine hydrochloride (60 mg/kg) and xylazine hydrochloride (5 mg/kg). Anaesthetized rats were sequentially placed on the operating table. The neck region was shaved. Through a midline ventral neck incision, the right and left carotid arteries were carefully isolated from the vagus nerve and surrounding tissues. Bilateral carotid artery ligation was performed in the transient ischemia–reperfusion and ischemia–reperfusion + DiOHF groups. In the sham group, the procedure was limited to isolating the right and left carotid arteries from the vagus nerve and surrounding tissues. After the surgical procedure, the animals were kept covered and maintained at 35 °C for 30 min using heating pads connected to a DC Temperature Controller System (FHC, USA). At the end of 30 min, the ligatures were removed to achieve reperfusion. Finally, the incision in the neck region was sutured (Aladag et al. 2024; Caliskan et al. 2016).

### Tissue preparation

Before homogenization, tissue weights were recorded. The tissues were then cut into small pieces and placed into glass test tubes. PBS (phosphate-buffered saline, pH 7.4) was added in an amount nine times the tissue weight. The samples were homogenised at 4 °C using a Misonix Microscan ultrasonic tissue homogeniser, and the resulting homogenates were centrifuged at 5000 × g for 5 min. The supernatants were then transferred into clean tubes.

### RNA isolation from striatal tissue

Total RNA isolation was performed in 28 rats to determine the expression levels of ICAM, calbindin, alpha-tubulin, and beta-tubulin genes using the real-time PCR method. A commercial RNA isolation kit (Bio Basic; Canada) was used for this procedure.

### cDNA synthesis

A commercial cDNA synthesis kit (OneScript Plus, ABM; Canada) was used to reverse transcribe the mRNA contained in the isolated total RNA into complementary DNA (cDNA). Equal amounts of RNA from each sample were used, and the steps in the manufacturer’s recommended protocol were followed sequentially (Table 1).

**Table 1:** cDNA synthesis mixture used to transcribe cDNA from mRNA

Component	Amount
5X RT Buffer	4 µl
dNTP	1 µl
Primer	1 µl
OneScript Plus RTase	1 µl
Nuclease-free H₂O	3 µl

For each sample, 10 µl of the reaction mixture were taken, and RNA was added. After mixing the tubes, the reverse transcription program was initiated using a Thermal Cycler (Bio-Rad, USA) according to the protocol specified in the kit. The reverse transcription program consisted of cDNA synthesis at 55 °C for 15 min, denaturation at 85 °C for 5 min, and holding at + 4 °C. The concentration and purity of the obtained cDNA were determined using a Nanodrop (SMA 1000, Merinton, China). The samples were then stored at –20 °C until the quantitative polymerase chain reaction (qPCR) process.

### Expression levels of ICAM, calbindin, alpha-tubulin, and beta-tubulin genes

The expression levels of ICAM, Calbindin, alpha-Tubulin, and beta-Tubulin genes were analyzed in a total of 28 rats using real-time PCR. During the preparation of the Real-Time PCR reactions, serial dilutions (1/1, 1/2, 1/4, 1/8, 1/16, and 1/32) were prepared from the cDNA obtained from the control group tissues in order to generate standard curves. Real-Time PCR was performed, and standard curves were constructed. Temperature ranges were tested to ensure optimal primer binding and achieve standardisation.

Primers used for the mRNA expression analysis of the target genes were synthesised by Sentebiolab (Turkey). For the reference gene, the oligomer GAPDH (glyceraldehyde-3-phosphate dehydrogenase) produced by a local Turkish company was used. Approximately 200 nanograms of primer were used for each reaction (Table 2).

**Table 2:** Primers used for real-time PCR

Gene	Primer Sequence	Function
ICAM Forward	5'-GTATCCATCCATCCCACACAGAAG −3'	Target Gene
ICAM Reverse	5'-CAGTTGTGTCCACTCGATAGTT −3'	Target Gene
Calbindin Forward	5'-ATTTCGACGCTGACGGAAGT-3'	Target Gene
Calbindin Reverse	5'-AGGTGATAACTCCAATCCAGCC-3'−3'	Target Gene
Beta Tubulin Forward	5'-GCAACCAGATCGGGGCCA-3'	Target Gene
Beta Tubulin Reverse	5'-GTTCATGATGCGGTCGGGAT-3'	Target Gene
Alpha Tubulin Forward	5'-GGAGCTCTACTGCCTGGAACAT-3'	Target Gene
Alpha Tubulin/Reverse	5'-CAATAACTGTGGGTTCCAGGTCTAC-3'	Target Gene
GAPDH Forward	5'-GGGCCAAAAGGGTCATCATC-3'	Reference Gene
GAPDH Reverse	5'-AACCTGGTCCTCAGTGTAGC-3'	Reference Gene

A commercial PCR kit (BlasTaq 2X Quantitative Polymerase Chain Reaction Master Mix, ABM, Canada) was used, and PCR reactions were carried out in a total volume of 20 µl. The reaction mixture contained 10 µL of Master Mix (enzyme, dNTPs, Mg, buffer, and water), 5 µL of cDNA, 1 µL each of forward and reverse primers, and 3 µL of nuclease-free water. After primer optimisation, the PCR program was set as follows: enzyme activation at 95 °C for 3 min, denaturation at 95 °C for 10 s, annealing and polymerisation at 60 °C for 1 min, for a total of 40 cycles. Reactions were performed using the CFX96 Touch™ Real-Time PCR Detection System (Bio-Rad, USA), and the results of the quantitative polymerase chain reaction were evaluated using the 2^−ΔΔCT^ method.

### ELISA analysis

The BDNF protein levels in striatal tissues obtained from 28 rat were determined using a commercial ELISA kit (Bioassay Technology/BT Laboratory, China; catalogue number E0476Ra). The kit’s standard curve range was 0.05–10 nanograms per millilitre. Sensitivity: 0.01 ng/mL. Intra-assay CV: < 8%. Inter-assay CV: < 10%. An ELISA reader from BMG Labtech (Germany) was used.

### Histological examination

Histological examination was performed on 28 rat-derived striatum tissues, which were freshly prepared and cooled at + 4 °C in 4% paraformaldehyde (PFA) fixative (fixative-to-tissue volume ratio of 10:1). The tissues were left at + 4 °C for 24 h. For cryomatrix embedding, tissues were placed into 30% sucrose and left for at least 24 h until the tissue sank. From tissues embedded in a cryomatrix, serial sections of 5 µm thickness were cut using a cryostat (Thermo Shandon Cryostat 210160 GB). For immunofluorescent labelling, sections were incubated in PBS containing 5% BSA (bovine serum albumin) and 0.2% Triton X-100 (polyethene glycol p-(1,1,3,3-tetramethylbutyl)-phenyl ether) for 20 min at room temperature. They were washed three times in PBS, each wash lasting 5 min. Excess liquid was aspirated. A protein blocking solution was applied and incubated for 30 min at 37 °C. The blocking solution was removed by pipette without washing. The primary antibody (Anti-NeuN Antibody, ab279297) was incubated for 1 day at + 4 °C. At the end of the day, sections were washed three times in PBS (5 min each) and then treated with the secondary antibody Goat anti-Rabbit IgG (H + L), FITC (656,111). After a 3-h incubation at room temperature, sections were washed three times in PBS (5 min each). Sections were mounted with DAPI-containing mounting medium. Procedures were performed in a dark, humidity-controlled chamber. Using an Olympus BX51 trinocular fluorescence microscope, fields were examined at 4x, 10x, 20x, and 40 × magnifications; for each magnification, four random fields were selected at 40x, and images were recorded with a DP72 camera. The total cell count in the field was determined by DAPI nuclear staining. The ratio of anti-NeuN labelled cells to total cells in the recorded images was calculated using the ImageJ software (National Institutes of Health, Bethesda, MD, USA).

### Statistical analysis

Statistical analyses were conducted using the SPSS 26.0 software package. For each parameter, arithmetic means and standard deviations were calculated. The Shapiro–Wilk test was employed to assess the normality of data distribution, confirming that the data followed a normal distribution. One-way analysis of variance (ANOVA) was applied to evaluate differences among groups, and post hoc comparisons were performed using Duncan’s multiple range test to identify the specific sources of significant variation. Statistical significance was defined as a p-value below 0.05.

## Results

### Calbindin gene expression findings

**Fig. 1:**
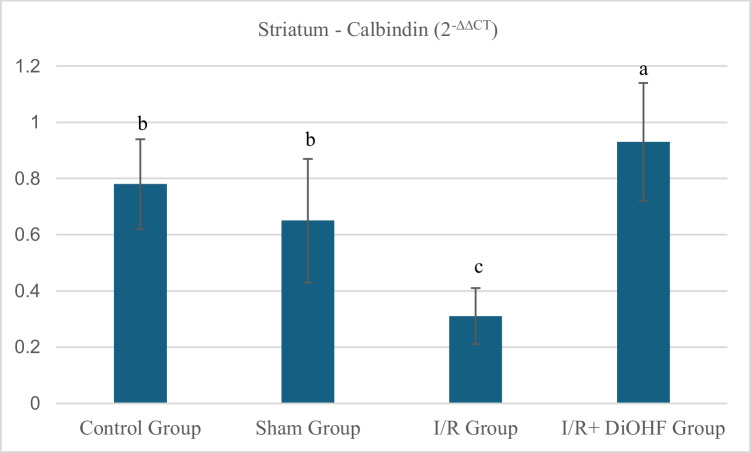
Expression level of the calbindin gene in striatum tissue across experimental groups. The expression level of the calbindin gene in the striatal tissue decreased with ischemia. DiOHF supplementation halted the decrease in expression level. There is a statistically significant difference among the different letters within the same column (P < 0.05; a > b > c). Control (n = 6); Sham (n = 6); I/R (n = 8); I/R + DiOHF (n = 8)

The calbindin levels in the striatal tissue of the groups are presented in Table 3 and Fig. 1 When the groups were compared, it was observed that the expression of calbindin in the striatum tissue showed a significant decrease following transient ischemia/reperfusion (I/R). However, in the I/R + DiOHF group, DiOHF supplementation was found to prevent the reduction in calbindin gene expression and to cause a significant increase in its expression level (P < 0.05).The expression level of the calbindin gene in the striatal tissue decreased with ischemia. DiOHF supplementation halted the decrease in expression level. There is a statistically significant difference among the different letters within the same column (P < 0.05; a > b > c) (Table 3).

**Table 3:** Expression level of the calbindin gene in the striatum

Groups	Mean ± SD
Control Group	0,78 ± 0,16 b
Sham Group	0,65 ± 0,22 b
I/R Group	0,31 ± 0,10 c
I/R + DiOHF Group	0,93 ± 0,21 a

The expression level of the calbindin gene in the striatal tissue decreased with ischemia. DiOHF supplementation halted the decrease in expression level. There is a statistically significant difference among the different letters within the same column (P < 0.05; a > b > c)

### Findings of alpha-tubulin gene expression

**Fig. 2:**
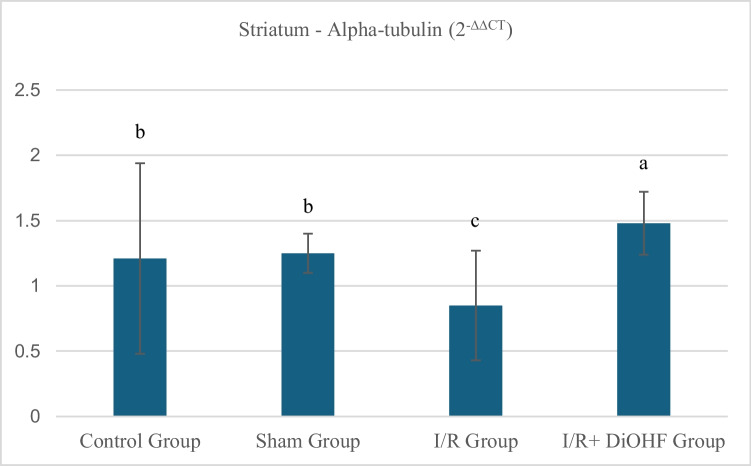
Expression level of the alpha-tubulin gene in striatum tissue across experimental groups. The expression level of the alpha-tubulin gene in striatal tissue decreased with ischemia. DiOHF supplementation halted the decrease and increased expression levels. There is a statistically significant difference among the different letters within the same column (P < 0,05; a > b > c). Control (n = 6); Sham (n = 6); I/R (n = 8); I/R + DiOHF (n = 8)

The expression levels of the alpha tubulin gene in the striatal tissue of the experimental groups are presented in Table 4 and Fig. 2 According to the data obtained from the experimental groups, the expression level of the alpha tubulin gene in the striatum tissue showed no significant difference between the control and sham groups. However, a significant decrease was observed in the transient I/R group, whereas a marked increase in expression values was detected in the I/R + DiOHF group, likely due to DiOHF supplementation (Table 4; P < 0.05). The expression level of the alpha-tubulin gene in striatal tissue decreased with transient ischemia-reperfüzyon. DiOHF supplementation halted the decrease and increased expression levels. There is a statistically significant difference among the different letters within the same column (P < 0,05; a > b > c).

**Table 4:** Expression level of the alpha-tubulin gene in the striatum

Groups	Mean ± SD
Control Group	1,21 ± 0,73 b
Sham Group	1,25 ± 0,15 b
I/R Group	0,85 ± 0,42 c
I/R + DiOHF Group	1,48 ± 0,24 a

The expression level of the alpha-tubulin gene in striatal tissue decreased with ischemia. DiOHF supplementation halted the decrease and increased expression levels. There is a statistically significant difference among the different letters within the same column (P < 0,05; a > b > c)

### Findings of beta-tubulin gene expression

**Fig. 3:**
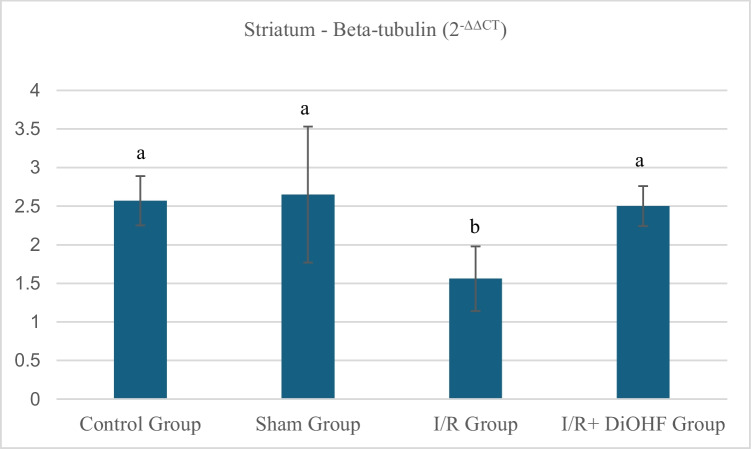
Expression level of the beta-tubulin gene in striatum tissue across experimental groups. Beta-tubulin gene decreased due to ischemia. DiOHF treatment prevented the decrease in expression. An increase in expression level was observed in the striatum tissue with DiOHF supplementation. There is a statistically significant difference between different letters in the same column (P < 0.05; a > b >). Control (n = 6); Sham (n = 6); I/R (n = 8); I/R + DiOHF (n = 8)

The beta tubulin values in the striatal tissue of the experimental groups are presented in Table 5 and Fig. 3 According to the data obtained from the experimental groups, the expression level of the beta tubulin gene was found to be similar in the control and sham groups, while a decrease was observed in the transient I/R group. However, in the I/R + DiOHF group, DiOHF supplementation prevented this reduction and resulted in a notable increase in the expression level (Table 5; P < 0.05). The expression level of the beta-tubulin gene decreased duewith transient ischemia–reperfusion. DiOHF treatment prevented the decrease in expression. An increase in expression level was observed in the striatum tissue with DiOHF supplementation. There is a statistically significant difference between different letters in the same column (P < 0.05; a > b >).

**Table 5:** Expression level of the beta-tubulin gene in the striatum

Groups	Mean ± SD
Control Group	2,57 ± 0,32 a
Sham Group	2,65 ± 0,88 a
I/R Group	1,56 ± 0,42 b
I/R + DiOHF Group	2,5 ± 0,26 a

The expression level of the beta-tubulin gene decreased due to ischemia. DiOHF treatment prevented the decrease in expression. An increase in expression level was observed in the striatum tissue with DiOHF supplementation. There is a statistically significant difference between different letters in the same column (P < 0.05; a > b > c)

### Findings of ICAM gene expression

**Fig. 4:**
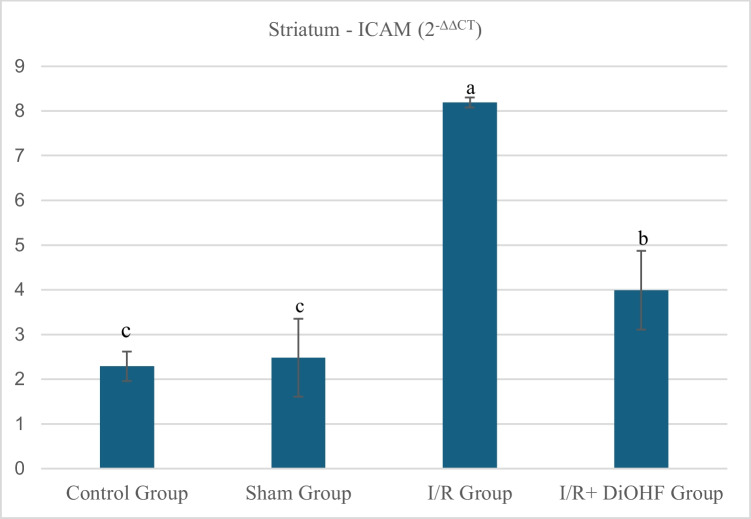
Expression level of the ICAM gene in striatum tissue across experimental groups. Ischemia increased the expression level of the ICAM gene in the striatum tissue. DiOHF supplementation prevented this increase. There is a statistically significant difference between different letters in the same column (P < 0.05; a > b > c). Control (n = 6); Sham (n = 6); I/R (n = 8); I/R + DiOHF (n = 8)

The ICAM values in the striatal tissue of the experimental groups are presented in Table 6 and Fig. 4 According to the data obtained from the experimental animals, the expression level of the ICAM gene in the striatal tissue showed no significant difference between the control and sham groups. However, an increase in expression was observed in the transient I/R group, while DiOHF supplementation in the transient I/R + DiOHF group suppressed this increase (Table 6; P < 0.05; a > b > c). Ischemia increased the expression level of the ICAM gene in the striatum tissue. DiOHF supplementation prevented this increase. There is a statistically significant difference between different letters in the same column (P < 0.05; a > b > c).

**Table 6:** Expression level of the ICAM gene in the striatum

Groups	Mean ± SD
Control Group	2,29 ± 0,33 c
Sham Group	2,48 ± 0,87 c
I/R Group	8,19 ± 0,11 a
I/R + DiOHF Group	3,99 ± 0,88 b

Ischemia increased the expression level of the ICAM gene in the striatum tissue. DiOHF supplementation prevented this increase. There is a statistically significant difference between different letters in the same column (P < 0.05; a > b > c)

### Findings of BDNF protein levels

**Fig. 5:**
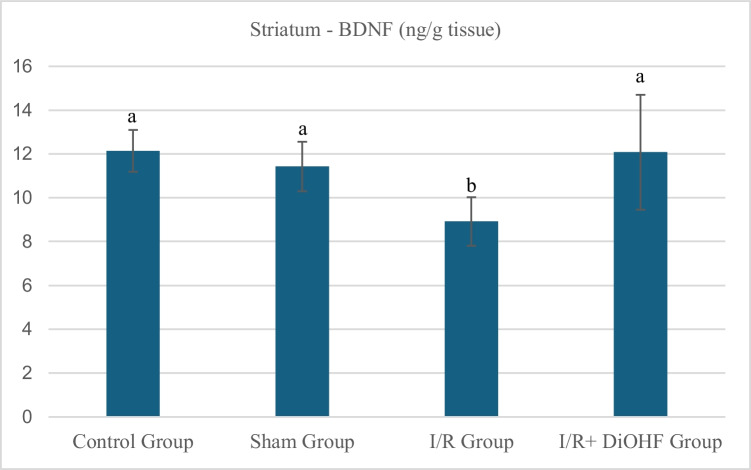
Level of the BDNF protein in striatum tissue across experimental groups. BDNF protein in the striatum tissue decreased with ischemia. DiOHF supplementation prevented the decrease and restored the level. There is a statistically significant difference between different letters in the same column (P < 0.05; a > b >). Control (n = 6); Sham (n = 6); I/R (n = 8); I/R + DiOHF (n = 8)

The BDNF values in the striatal tissue of the experimental groups are presented in Table 7 and Fig. 5 According to the data obtained from the animals in the experimental groups, the level of the BDNF protein in the striatal tissue was similar between the control and sham groups. However, a decrease in level was observed in the I/R group. With DiOHF supplementation, this decrease was prevented, and the level in the I/R + DiOHF group was restored to a level close to that of the control group (Table 7; P < 0.05). The level of the BDNF protein in the striatum tissue decreased with ischemia. DiOHF supplementation prevented the decrease and restored the level. There is a statistically significant difference between different letters in the same column (P < 0.05; a > b >).

**Table 7:** Level of the BDNF protein in the striatum

Groups	Mean ± SD
Control Group	12,14 ± 0,95 a
Sham Group	11,43 ± 1,13 a
I/R Group	8,92 ± 1,11 b
I/R + DiOHF Group	12,08 ± 2,62 a

The level of the BDNF protein in the striatum tissue decreased with ischemia. DiOHF supplementation prevented the decrease and restored the level. There is a statistically significant difference between different letters in the same column (P < 0.05; a > b > c)

### Anti-NeuN antibody labelling findings

**Fig. 6:**
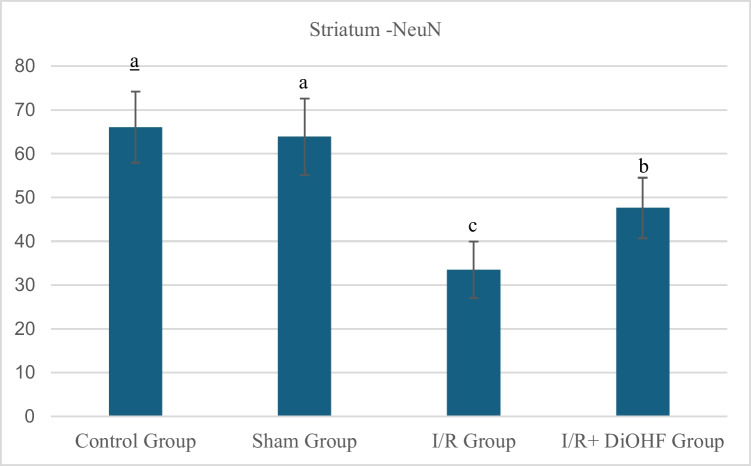
Fluorescent signal indicator of NeuN protein staining in striatum tissue across experimental groups. Fluorescent signal in the striatum tissue decreased in the ischemia group. DiOHF supplementation prevented the decrease and led to an increase. There is a statistically significant difference between different letters in the same column (P < 0.001; a > b > c). Control (n = 6); Sham (n = 6); I/R (n = 8); I/R + DiOHF (n = 8)

**Fig. 7:**
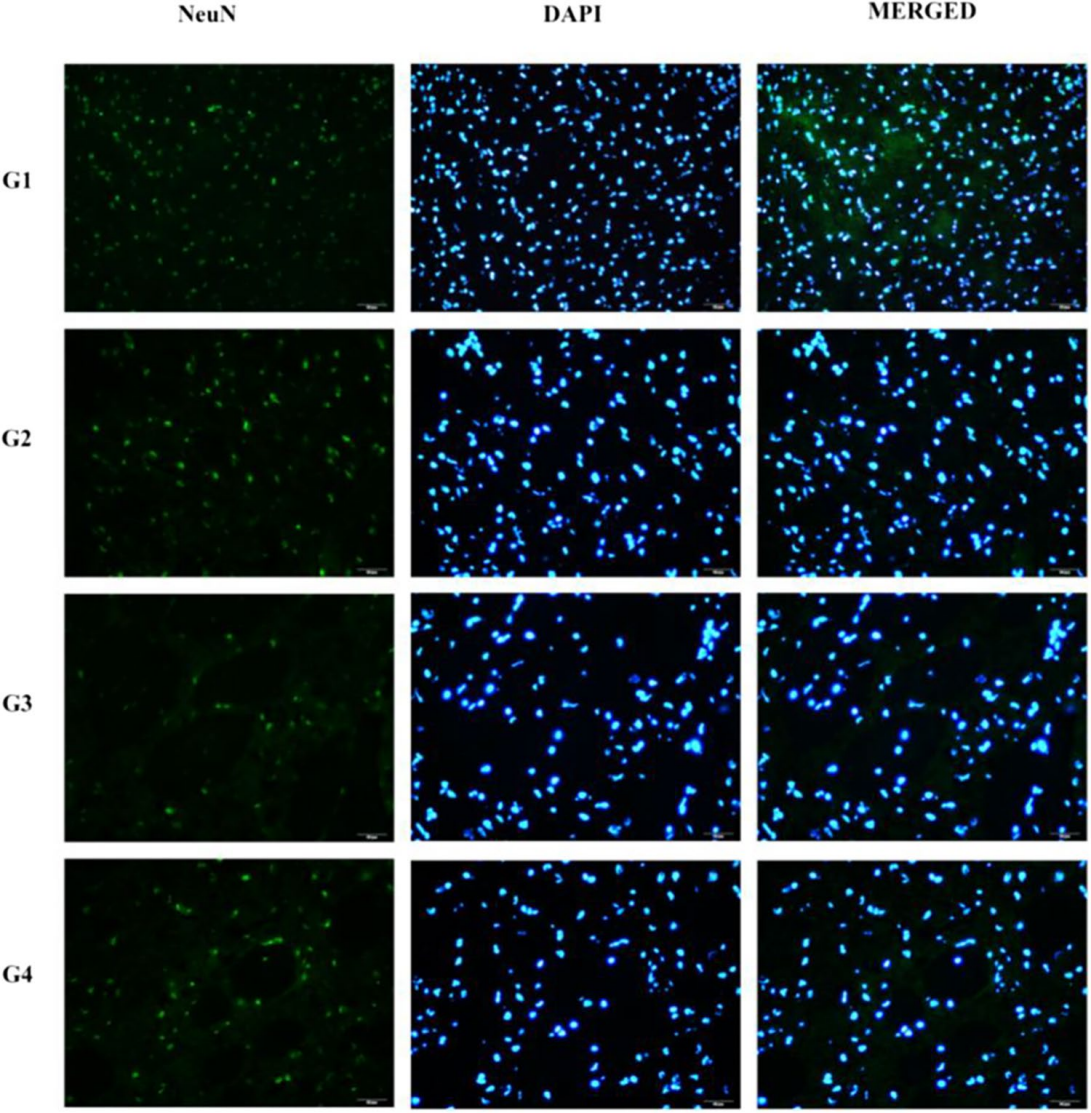
NeuN staining of the striatum. Fluorescent images obtained by immunolabeling to demonstrate neurogenesis in the striatum. The DAPI column represents all nucleated cells in the striatum; the NeuN column indicates mature neurons in the striatum. The merged DAPI and NeuN images are shown in the merged column. Arrows indicate mature neurons as examples (× 40 magnification). Control (n = 6); Sham (n = 6); I/R (n = 8); I/R + DiOHF (n = 8)

The fluorescent signal indicator of NeuN protein staining.in the striatal tissue of the experimental groups are presented in Table 8 and Figs. 6 and 7. According to the data obtained from the experimental groups, the fluorescent signal indicator of NeuN protein staining in the striatal tissue were similar between the control and sham groups. A significant decrease was detected in the transient I/R group. However, an increase was observed again following DiOHF supplementation (P < 0.001). The fluorescent signal indicator of NeuN protein staining in the striatum tissue decreased in the transient ischemia–reperfusion group. DiOHF supplementation prevented the decrease in fluorescent signal level and led to an increase. There is a statistically significant difference between different letters in the same column (P < 0.001; a > b > c). (Table 8 and Fig. 1; P < 0.05).

**Table 8:** Fluorescent signal indicator of NeuN protein stainingin the striatum

Groups	Mean ± SD
Control Group	66,08 ± 8,13 a
Sham Group	63,87 ± 8,74 a
I/R Group	33,48 ± 6,47 c
I/R + DiOHF Group	47,61 ± 6,91 b

Fluorescent signal indicator of NeuN protein staining in the Striatum

tissue decreased in the ischemia group. DiOHF supplementation prevented the decrease and led to an increase. There is a statistically significant difference between different letters in the same column (P < 0.001; a > b > c)

## Discussion

In the present study, we investigated the effects of 3′,4′-dihydroxyflavonol (DiOHF), a synthetic flavonol, on inflammatory and neurotrophic signaling pathways in a rat model of transient cerebral ischemia–reperfusion (I/R) induced by bilateral common carotid artery occlusion. Our findings demonstrate that bilateral carotid occlusion induced transient ischemia–reperfusion markedly altered the expression of neuronal survival marker, BDNF, and ICAM, and tubulin in the striatum, whereas 7-day DiOHF treatment partially ameliorated these changes, suggesting a neuroprotective and modulatory role for DiOHF in ischemia-induced brain injury.

When the results of the present study are evaluated as a whole, it is observed that experimental bilateral carotid occlusion induced transient cerebral ischemia–reperfusion in rats led to decreases in calbindin, tubulin, BDNF, and NeuN levels and an increase in ICAM levels in the striatal tissue. However, it was determined that one week of DiOHF supplementation following transient ischemia–reperfusion led to significant improvements in the adverse effects caused by I/R.

Previous studies have investigated the relationship between flavonoids and calbindin. Notably, while amphetamine (Adderall) acts as a central nervous system stimulant, luteolin possesses neuroprotective activity. In rats treated with Adderall, glial fibrillary acidic protein (GFAP), ionised calcium-binding adapter molecule 1 (Iba1), and anti-calbindin expression levels were increased in the cerebral cortex. However, daily oral administration of luteolin for four weeks restored all these parameters toward control values, with the higher dose being more effective than the lower one (Koriem and El-Soury 2024). In another study, quercetin supplementation was shown to reduce cerebellar apoptosis and gliosis while increasing calbindin levels (Abdelrahman et al. 2023). Similarly, the combined administration of bazedoxifene and genistein combination led to improvements in hippocampal neurodegeneration by increasing the neuronal expression of calbindin, BDNF, Trk-B, and ERK (Samak et al. 2023). Genistein, a soy isoflavone, is structurally and functionally similar to endogenous estrogen, possessing the potential to mimic estradiol activity. Treatment of hens with genistein resulted in a significant upregulation of calbindin-D28k and transient receptor potential vanilloid channel type 6 (TRPV6) mRNA expression in the shell gland (Saberifar et al. 2021). In another study, hesperidin and chrysin could demonstrated a significant increase in calbindin-D28k immunopositivity following hesperidin and chrysin treatment (Hanedan et al. 2018). In the present study, the effect of the flavonoid DiOHF on calbindin expression was investigated for the first time, as no previous studies have examined this relationship.

We also evaluated tubulin, in which showed a decrease in the striatum (I/R), whereas DiOHF treatment resulted in a significant increase following transient ischaemia–reperfusion. In a comparative study of 24 flavonoids, the cytotoxic and antiangiogenic activities on B16 and Lewis lung carcinoma cells and endothelial cells were assessed. The most active agent, fisetin, exhibited a unique cell morphology compared to colchicine, combretastatin A-4, docetaxel, and cytochalasin D. Resistance to cold depolymerisation and a 2.4-fold increase in acetylated α-tubulin suggested that fisetin acts as a microtubule stabiliser (Touil et al. 2009). Ischemic cerebrovascular disease promotes activation and differentiation of neural stem cells (NSCs) into mature neurons and glial cells to repair neural damage. Both in vivo and in vitro Astragalus flavone enhanced β-tubulin III expression in cerebral infarction models (Gao et al. 2022).

Another study investigated whether epigallocatechin gallate (EGCG) protects retinal neurons from ischemia/reperfusion injury and oxidative stress induced by hydrogen peroxide in vitro. Following 45 min of ischemia and 7 days of reperfusion, tubulin levels in the optic nerves of vehicle-treated eyes decreased by 47%, while EGCG treatment partially prevented this reduction (Zhang et al 2007). In the present study, the synthetic flavonoid DiOHF demonstrated a protective effect by preventing the reduction in tubulin levels in striatal tissue following transient bilateral carotid occlusion induced cerebral I/R, which is consistent with previous findings on other flavonoids showing similar tubulin-stabilising effects under ischemic conditions.

Another parameter analysed in this study was the intercellular adhesion molecule (ICAM). ICAM plays a crucial role in vascular adhesion that occurs as a result of blood flow deceleration. In our study, ICAM expression increased in the striatum following transient I/R, whereas DiOHF treatment markedly reduced its levels. Previous studies have reported that flavonoids modulate ICAM expression and inflammation in various I/R models. For example, fisetin exhibited neuroprotective effects in ischemic brain injury by inhibiting inflammatory mediators, including ICAM-1, through modulation of NF-κB signalling (Zhang and Cui 2021). Similarly, Rabdosia rubescens total flavonoids reduced ICAM-1 and serum S-100β levels in rat models of focal cerebral I/R, indicating protective vascular and anti-inflammatory properties (Miao et al. 2017). Furthermore, scutellarin ethyl ester significantly decreased ICAM-1 expression in rats subjected to middle cerebral artery occlusion (MCAO) (Ma et al. 2017). Methylophiopogonanone A, an active homoisoflavonoid from *Ophiopogon japonicus*, was shown to inhibit ICAM-1 and VCAM-1 expression and leukocyte-endothelial adhesion (Lin et al. 2015). Additionally, Hydroxysafflor Yellow A prevented cerebral I/R injury by inhibiting thrombin formation and suppressing both ICAM-1 expression and neutrophil infiltration (Sun et al. 2010). Likewise, taxifolin reduced cerebral infarction and inhibited Mac-1 and ICAM-1 expression, key adhesion receptors mediating leukocyte–endothelial interactions (Wang et al. 2006). Finally, hydroxyethylpuerarin significantly suppressed myeloperoxidase activity and ICAM-1 expression following 2 h of ischemia and 24 h of reperfusion (Lou et al. 2004). Consistent with these reports, the present study is the first to demonstrate that DiOHF significantly reduces ICAM expression in an experimental transient I/R model, suggesting its potential neuroprotective mechanism.

The present study also examined BDNF (Brain-Derived Neurotrophic Factor). Following transient I/R, BDNF levels decreased significantly in the striatum. Previous studies have shown that striatal neurons depend on neurotrophins, particularly BDNF, for development, survival, and proper function (Baydyuk and Xu 2014). As the striatum does not produce BDNF endogenously, abnormalities in anterograde transport or reduced expression in BDNF-supplying brain regions can cause neuronal dysfunction and striatal atrophy (Baydyuk and Xu 2014). Experimental gene transfer using adeno-associated viral vectors demonstrated that ectopic BDNF expression in the striatum enhances neuronal differentiation of transplanted progenitor cells, while FGF2 expression promotes their survival and proliferation (Chen et al. 2007).

Flavonoids are also known to increase BDNF expression via activation of ERK and Akt signalling pathways, thereby enhancing neuronal connectivity and plasticity (Manach et al. 2004). Consistent with these findings, our study showed that DiOHF treatment reversed the I/R-induced suppression of BDNF, supporting the view that flavonoids enhance BDNF-mediated neuroprotection.

In the present study, the effects of bilateral carotid occlusion induced transient cerebral ischemia/reperfusion (I/R) and subsequent DiOHF treatment on NeuN immunoreactivity in striatal tissue were also evaluated.NeuN activity in the striatum was suppressed by I/R, whereas DiOHF treatment prevented this inhibition.NeuN immunoreactivity is a specific marker for neurons, and a reduction in the number of NeuN-positive cells under pathological conditions is generally considered evidence of neuronal loss. However, the decrease in NeuN labelling may also result from protein depletion or loss of antigenicity. Therefore, after cerebral ischemia, the morphological characteristics of neurons that lost NeuN immunoreactivity and NeuN protein levels were investigated in the mouse brain. The number of NeuN-labelled cells in the penumbral and core regions decreased 6 h after mild ischemic injury (30 min of middle cerebral artery occlusion) (Ünal-Çevik et al. 2004).

In contrast to patterns of focused middle cerebral artery features, transient bilateral carotid shapes do not form a sharply demarcated infarction. Instead, they are caused by diffuse cerebral hypoperfusion and delayed lateral stress; with the hippocampus being the most vulnerable structure, additional trophic segments extending to other forebrain regions, including the striatum. Therefore, striatal changes observed in this way should be interpreted as indicative of distribution dysfunction caused by selective forebrain hypoperfusion, rather than tissue distribution provided by focused infarction. In our study, one week of DiOHF supplementation following transient ischemia/reperfusion (I/R) significantly enhanced neuronal survival in which had been suppressed by I/R in striatum, showing similarities with findings reported for other flavonoids. However, no significant difference was observed between striatum tissue in terms of the number of active cells per unit area. This suggests that the drug and treatment dose used may exert comparable effects in striatum tissue.

## Conclusion

The present findings demonstrate that transient bilateral carotid occlusion followed by reperfusion, induces marked deterioration in neuronal phenotype-associated, neurotrophic, and inflammatory markers in striatal tissue. Although this model does not represent focal territorial infarction, it produces sufficient forebrain ischemic stress to impair neuronal molecular integrity. Subsequently, the levels of calbindin, tubulin, and BDNF, which are key molecules in brain function, were found to decrease following I/R, while ICAM levels in the striatum tissue increased, indicating the occurrence of I/R-induced injury. Moreover, NeuN activity, serving as an indicator of mature neuronal marker was suppressed in the striatum tissue. One week of DiOHF treatment substantially ameliorated these alterations, supporting a protective role of DiOHF against hypoperfusion/reperfusion-induced neuronal injury.

## Study limitation

This study has several limitations that should be considered when interpreting the results. This study investigated the effects of DiOHF in a rat model of transient global cerebral ischemia–reperfusion (I/R) induced by bilateral common carotid artery occlusion.The investigation was limited to biochemical assessments, specifically ELISA-and PCR based quantification of examined parameters evaluations such as or gene expression profiling. The absence of such multimodal approaches restricts our ability to correlate biochemical changes with structural or functional outcomes in the brain. In future large-scale studies, a detailed elucidation of the molecular mechanisms involved in these pathways is required to better understand the effects of these markers and to establish potential therapeutic targets for different ischemia–reperfusion conditions.Mature neuronal marker Neu N in this study was evaluated in striatal tissue, rather than through region-specific analyses of classical neurogenic niches such as the subgranular zone (SGZ) and subventricular zone (SVZ).

It is essential to distinguish this transient global ischemia model from focal models like MCAO. While focal models result in a territorial infarct, our tBCAO model induces selective neuronal vulnerability, particularly affecting regions like the hippocampus and the striatum.

## Data Availability

The datasets in which were generated during the current study are available from the corresponding author upon reasonable request.
